# Public trust in the long-term care insurance pilot program in China: An analysis of mediating effects

**DOI:** 10.3389/fpubh.2022.928745

**Published:** 2022-07-19

**Authors:** Rong Peng, Wansha Zhang, Xueqin Deng, Bei Wu

**Affiliations:** ^1^Institute of New Development, Guangdong University of Finance and Economics, Guangzhou, China; ^2^Rory Meyers College of Nursing, New York University, New York City, NY, United States

**Keywords:** long-term care insurance, public trust, insurance awareness, public satisfaction, mediating effect

## Abstract

**Objective:**

This study aimed to evaluate the implementation of the long-term care insurance (LTCI) pilot program in China through an examination of public trust in the system and its associated factors of insurance awareness and satisfaction with the LTCI policy.

**Method:**

An online survey was used to collect data from 786 participants in the city of Guangzhou, one of the pilot sites of the LTCI. Ordinal logistic regression models were used to investigate the related factors of public trust in the LTCI. Structural equation modeling (SEM) was conducted to test the mediating effect of satisfaction with LTCI policy on the relationship between insurance awareness and public trust.

**Results:**

More than 60% of participants gave a positive evaluation of the LTCI pilot program. More than 70% of the participants recognized the important role of the program. Both an understanding of insurance and satisfaction with LTCI policies were associated with public trust. The proportion of the indirect effect with regard to the total effect of satisfaction on trust was 70.133%, greater than the direct effect of 29.867%.

**Conclusions:**

Our findings supported the hypothesis that satisfaction with the LTCI policy plays a mediating role between insurance awareness and public trust. Optimalization of the LTCI policy was recommended to improve public trust in the LTCI program.

## Introduction

The aging of the population poses a daunting challenge to the long-term care system in China. There were 52.71 million Chinese older adults with disabilities in 2020 and this number was projected to increase to 136.74 million by 2030 (1). It is estimated that the total economic costs of long-term care in China will increase from $68.69 billion dollars in 2020 to $246.76 billion (measured in 2010 US Dollars) by 2050 (2). Moreover, there are a significant number of older adults with disabilities that lack financial support and services for long-term care (3, 4).

China introduced a long-term care insurance (LTCI) program for older adults as a means to meet these challenges. In 2016, the Chinese pilot program was initiated in 15 cities (5) and expanded to include 14 additional sites in 2020 (6). According to the Chinese National Healthcare Security Administration, the number of LTCI enrollees in 15 pilot cities had reached 110 million in 2020 (7). Approximately 1.36 million people have received an LTCI benefit (7). The Chinese central government encouraged local governments to implement LTCI pilot programs in the context of local economy and population structure. Thus, there are significant disparities in the provision of LTCI benefits and long-term care services across these pilot sites. For example, several studies highlighted gaps in insurance coverage, eligibility, funding sources, and care provision (8, 9).

Several studies were conducted to evaluate the performance of the LTCI implementation in China. These studies found that China's LTCI system had significantly reduced beneficiaries' medical expenses (10) and the likelihood of older adults reporting unmet long-term care needs (11). People are willing to support an expansion of the formal implementation of LTCI policy in China (12). Although these studies provide empirical evidence of the implementation effects of LTCI programs in China, there is a lack of studies on public trust and satisfaction with LTCI programs in China. There is a need to evaluate the impact of LTCI policy development from diverse perspectives and using various evaluation methods (9).

The aim of this study was to evaluate the implementation effect of the LTCI pilot program in China through an examination of public satisfaction and trust in the system. Specifically, this study analyzed the complex relationships among insurance awareness, satisfaction, and trust in the LTCI pilot program based on the pilot city Guangzhou. Guangzhou is different from some of the other pilot cities—such as Shanghai and Qingdao—because the city has a lower proportion of aging population and a higher level of economic development in comparison to most other participating sites (13). The pilot program in Guangzhou was initiated in 2017. Compared with most pilot cities that only cover the individuals with severe disability, Guangzhou's LTCI program has a broader coverage. It covers all disabled persons and those with moderate and severe dementia (13). The reimbursement rate is among the highest in the pilot cities. The basic daily care expenses are paid by the LTCI fund with a maximum of 120 Yuan (about 18 US Dollars) per day for institutional care and a maximum of 115 Yuan (about 17 US Dollars) per day for home care (9).

Our study contributes to the literature by providing evidence on the relationship among insurance awareness, satisfaction, and trust. First, public satisfaction with the healthcare system is one of the indicators used by the World Health Organization (WHO) to evaluate the quality of healthcare systems (14, 15). Customer satisfaction has been the most frequently recognized pathway toward sustainable competitive advantage (16). Satisfaction represents an important dimension of the quality of a program. Second, universal health insurance coverage policies should be tailored to individual's needs and expectations as recommended by the WHO (17). Trust in the program proves crucial to the goal of ensuring that the LTCI serves the participants' best interests. Third, public trust and satisfaction are central to the implementation of the LTCI pilots. Trust leads to collaborative behavior and participation (18, 19). The effort to expand the pilot sites to the construction of a formal LTCI structure, simultaneously provides an opportunity for people to gain confidence in the system. Individual's acceptance and support of the pilot programs would encourage their attendance, an important indicator of the program's success. Finally, residents' trust in the LTCI can provide evidence for policymakers to used it as an indicator of the program's performance. Both public trust and satisfaction reflect a community consensus about public policy (20). In the context of this study, public trust and satisfaction were used to evaluate the feasibility of the LTCI pilot programs in China. Therefore, the findings from this study will inform future initiatives designed to promote the LTCI pilot programs.

## Literature review and hypotheses

### Insurance awareness and satisfaction

One of the potential predictors of the enrollees' satisfaction was their awareness related to the object evaluated (21, 22). Health insurance awareness refers to individual's knowledge of the availability and coverage afforded through health insurance (23, 24), their willingness to demand and use it (25), and their expectations with regard to its coverage, role in their own wellbeing, and implementation (26). Because of the complexity of the insurance policies, the awareness of health insurance depends on both the enrollees' education level (27, 28) and the developments integral to the insurance industry (29). According to the customer satisfaction theory (30, 31), satisfaction is a subjective feeling after comparing the actual situation with personal expectations. It is an attitude that is often accompanied by values about the object being evaluated (30). As a new insurance program in China, the construction of LTCI is based on the current status of economic development and population aging (6, 9). The Chinese government has adopted a path from pilot testing to implementation in order to build a LTCI system at a national level (13). In the process of piloting, the public's knowledge and understanding of LTCI have gradually improved, forming psychological anticipations for the LTCI system. According to the expectation theory (32), the residents' satisfaction with the LTCI system fundamentally depends on their expectations of the LTCI system. Previous studies found that individuals' awareness of health insurance positively predicted individuals' satisfaction with health insurance (21, 28, 33). Therefore, our first hypothesis is as follows:

*Hypothesis 1: An individual's awareness of health insurance is positively associated with his/her satisfaction with the LTCI system*.

### Satisfaction and trust

Trust is a multidimensional concept that is comprised of competence and value congruence (34). It can be directed toward individuals, groups, organizations, or an operating system (35). Public trust in the public system is a basic factor in the operation of the public system and the efficiency of the health system (36). According to the customer perceived value theory (37), Higher satisfaction was associated with a positive value recognition of the object of evaluation and would result in greater trust (38). One study conducted in Indonesia indicated that patients' satisfaction had significant positive effects on trust in public hospitals (39). Similarly, satisfied insurance enrollees tend to trust their health insurance programs (21). Increased customer satisfaction results in an increase in the perceived value of insurance programs and an increased willingness to participate in public insurance plans (16). Based on the positive association between satisfaction and trust in previous studies (16, 21, 40), we proposed the second hypothesis:

*Hypothesis 2: The individuals' satisfaction with the LTCI is positively associated with their trust in the LTCI system*.

### Insurance awareness and trust

Previous studies found linkages between consumers' awareness and cognitive-based trust, a part of overall trust that is based on the knowledge (40). For example, consumers' awareness of food safety certification was associated with their trust in food safety (41). Individuals with knowledge about the HPV vaccine were more likely to have trust in cancer information (42). Awareness and knowledge often lead to recognition and/or acceptance of a new social program (43). Studies have shown that people who participate in insurance have more trust in the insurance system than those who do not participate in it (44).

Based on the findings from previous studies, we assume that awareness of health insurance is associated with trust in the LTCI program. Firstly, LTCI is a type of social insurance that has only recently been introduced in China. Due to the limited number of pilot sites, not everyone has the opportunity to learn about LTCI. An awareness of the insurance program would imply that individuals have obtained knowledge about it and enjoy an opportunity to consider its value (23, 25). Secondly, individuals value public policies and consider them to be fair when the information comes from a trusted institution (19). The central government stipulated that local governments should provide publicity to improve awareness of the LTCI policy. Awareness of the LTCI would increase recognition of the newly built LTCI health system. This would increase their trust or confidence in LTCI program. In addition, the relationships between awareness and satisfaction, and between satisfaction and trust, would suggest that an improved awareness of health insurance could encourage greater levels of trust through the indirect path of improved satisfaction. Several studies show that satisfaction was a valid mediator between trust and its associated factors such as perceived value (21) and service quality (16). Thus, we argued that the satisfaction with LTCI mediates the relationship between awareness and trust in the LTCI system. Consequently, we proposed Hypothesis 3 and 4:

*Hypothesis 3: An individual's awareness of LTCI is positively associated with their trust in LTCI system*.

*Hypothesis 4: Satisfaction is a mediator on the association between awareness and trust in the LTCI*.

## Materials and Methods

### Survey design

The questionnaire was designed to investigate the trust and satisfaction of Guangzhou residents on LTCI pilot. Privacy and confidentiality, the purpose of the study was stated at the beginning of the questionnaire, and the informed consent was completed prior to the data collection. The questionnaire included four sections: socio-demographic information, insurance awareness, satisfaction with the LTCI policy, and public trust. We found in the pilot survey that some residents did not understand the financing, payment and assessment procedures stipulated in the LTCI policy, on which we added explanations in the formal questionnaire.

The COVID-19 pandemic has forced many researchers to limit their work to online research. We conducted this cross-sectional survey through the *Wenjuanxing* platform from November 10 to 20, 2020. The *Wenjuanxing* platform is an open system that permits the collection of information through individual WeChat accounts (the most common social media in China). The platform has over 6.2 million registered users, and more than 10 million viewers every day. The platform provides the service of conducting surveys for specific targeted groups. It also ensures that only one submission may be received for each IP address. Participation was voluntary and anonymous. We employed the paid sampling service provided by the *Wenjuanxing* platform to send questionnaires to adult residents aged 18 and above in Guangzhou.

### Participants

During the specified period of investigation, Wenjuanxing issued an invitation to 1,000 eligible individuals (residents aged 18 years and above) to fill out a survey questionnaire, of which 816 completed. In order to ensure the quality of the data, we excluded the participants with invalid response. We included a total of 786 eligible participants in the study.

### Variables and measurement

#### Socio-demographic variable

Socio-demographic variables included gender (male = 0, female = 1), age (18–29 = 0, 30–44 = 1, 45 and older = 2), education level (below high school = 0, high school = 1, junior college = 2, bachelor's degree and above = 3), marital status (unmarried/divorced/widowed = 0, married = 1), health status (very bad = 0, bad = 1, fair = 2, good = 3, very good = 4), living arrangement (alone = 0, with others = 1), and income per month (<2,000 yuan = 0, 2,000–3,999 = 1, 4,000–5,999 = 2, 6,000–7,999 = 3, 8,000–9,999 = 4, 10,000+ = 5).

#### Response variable

The response variable of trust was a latent variable. After a thorough review of the literature, we adopted instruments from prior studies and used in our study. In a health insurance context, trust can be explained as enrollers' belief that they can rely on the insurance system. In this study, public trust was measured using two indicators adopted from Geng et al. (21) and Johnson and Grayson (40): willingness to provide a positive evaluation of the LTCI pilot program and perceived importance of the LTCI. The participants were asked the extent to which they would give a positive evaluation of the LTCI pilot program. The possible responses included very unwilling (0), unwilling (1), somewhat willing (2), willing (3) and very willing (4) by using a five-point Likert scale. Another question addressed how they would rate the importance of the LTCI system. Possible responses similarly ranged from very unimportant (0) to very important (4).

#### Explanatory variable

The explanatory variable of insurance awareness was measured using three items adopted from Reshmi et al. (24) and Kazaure (25). In this study, it reflected the evidence of commercial insurance awareness, social insurance awareness, and knowledge of the Guangzhou LTCI pilot. It included three indicators: the purchase of private insurance (No = 0, Yes = 1), participation in social medical insurance (No = 0, Yes = 1), and awareness of the LTCI pilot in Guangzhou (No = 0, Yes = 1).

#### Mediating variable

The mediating variable was satisfaction with the LTCI policies. We used three items modified from Geng et al. (21) and Liu et al. (44) to measure satisfaction with the LTCI policy. It indicates respondents' approval of the way that insurance funds were financed, reimbursement were paid, and the disability assessment procedures conducted. This variable included three satisfaction indicators: LTCI funding, LTCI payments, LTCI disability evaluations. Variables were scored on a four-point Likert scale that ranged from very dissatisfied (0) to very satisfied (4).

The information on the measurements of the latent variables including trust, insurance awareness and satisfaction are presented in the supplementary documents (Appendix, Supplementary Table S1). This self-constructed measuring tool for insurance awareness, satisfaction and trust, exhibited acceptable internal consistency with Cronbach's coefficient of 0.618, 0.723 and 0.634, respectively.

### Statistical analyses

Data management and statistical analysis were performed using SPSS version 17 and IBM SPSS Amos version 21. The level of significance was at *p* < 0.05. Descriptive analysis was conducted for the socio-demographics variables, insurance awareness, satisfaction with LTCI policy, and public trust. Categorical variables are presented as frequency and percentage. To establish the conditions necessary for the test of the mediation relationship (45), Chi-square test was employed to detect correlation of categorical variables. The ordinal logistic regression models were used to investigate the related factors of public trust in the LTCI. The dependent variable in model 1 was participant willingness to provide a positive evaluation of the LTCI pilot. The dependent variable in model 2 was the importance they gave to the LTCI system. Social-economic variables were included in both models.

**Figure 1:**
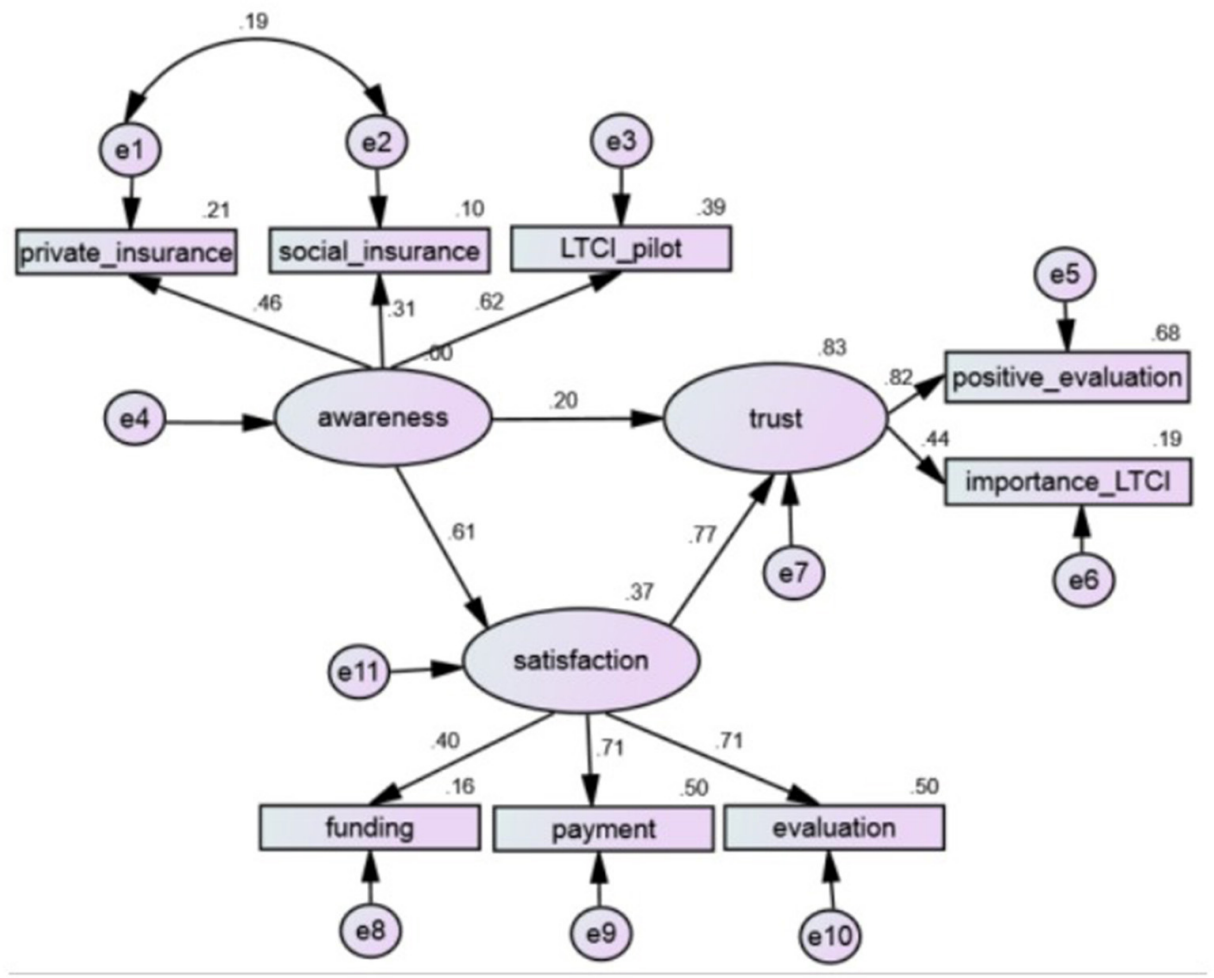
Structural equation modeling of satisfaction as a mediator of trust.

Structural equation modeling (SEM) was conducted to test the mediating effect of satisfaction with the LTCI policy in the relationship between insurance awareness and public trust. SEM has been widely used in healthcare services and outcomes research. For example, Geng et al. (21) used an SEM approach to assess patients' satisfaction with health insurance in China. A graphic presentation of the SEM model appears in Figure 1. A fully mediated relationship occurs when the influence of the explanatory variable occurs through the mediator, whereas a partially mediated relationship occurs when the influence of the explanatory variable is transmitted both as a direct effect by and an indirect effect through the mediator variable (45).

The Browne's asymptotically distribution-free method (46) was used to estimate parameters in the SEM model, considering that all observed variables were categorical and did not conform to the normal distribution. The bias-corrected percentile method was used to estimate the 95% confidence intervals of standardized coefficients. Bootstrapping with 2,000 random samples was used to test robustness of mediating effect (47). The model goodness-of-fit was evaluated by indices including a normed chi-square (equal to chi-square divided by its degree of freedom, values <5), the root mean square error of approximation (RMSEA, values <0.08), the Tucker-Lewis index (TLI, values > 0.95), and the comparative fit index (CFI, values > 0.95) (48).

## Results

### Description of the study sample

Table 1 shows the demographic characteristics of the respondents. The sample was comprised of 54.42% women and 47.58% men. More than 60% of respondents were under the age of 45, had a bachelor's degree or above, were married or cohabiting, and in good health. More than 80% of the respondents lived with others. The number of respondents with a monthly income of more than 6,000 yuan accounting for ~60%.

**Table 1:** Descriptive statistics of the survey sample (*N* = 786).

**Variables**			**Frequency (n)**	**Percentage (%)**
Socio-demographic variables	Gender	Female	412	52.42
		Male	374	47.58
	Age	18~29	241	30.66
		30~44	283	36.01
		45 or above	262	33.33
	Education level	Below high school	33	4.20
		High school	91	11.58
		Junior college	163	20.74
		Bachelor's degree or above	499	63.48
	Marital status	Unmarried/divorced/widowed	268	34.10
		Married	518	65.90
	Self-rated health status	Very bad	3	0.38
		Bad	40	5.09
		Fair	218	27.74
		Good	375	47.71
		Very good	150	19.08
	Living arrangement	Alone	121	15.39
		With others	665	84.61
	Income per month	<2,000 yuan	157	19.97
		2,000~3,999 yuan	54	6.87
		4,000~5,999 yuan	131	16.67
		6,000~7999 yuan	144	18.32
		8,000~9,999 yuan	152	19.34
		10,000+ yuan	148	18.83
Insurance awareness	Purchase of private insurance	No	239	30.40
		Yes	547	69.60
	Participating social insurance	No	169	21.50
		Yes	617	78.50
	Knowledge of LTCI pilot	No	427	54.33
		Yes	359	45.67
Satisfaction with LTCI policies	Satisfaction with funding	Very dissatisfied	5	0.64
		Dissatisfied	60	7.63
		Neither satisfied nor dissatisfied	208	26.46
		Satisfied	349	44.40
		Very satisfied	164	20.87
	Satisfaction with payment	Very dissatisfied	6	0.76
		Dissatisfied	43	5.47
		Neither satisfied nor dissatisfied	310	39.44
		Satisfied	359	45.67
		Very satisfied	68	8.65
	Satisfaction with disability evaluation	Very dissatisfied	6	0.76
		Dissatisfied	58	7.38
		Neither satisfied nor dissatisfied	287	36.51
		Satisfied	363	46.18
		Very satisfied	72	9.16
Trust in LTCI	Willingness to recommend the LTCI pilot	Very unwilling	5	0.64
		Unwilling	18	2.29
		Not so willing	271	34.48
		Willing	388	49.36
		Very willing	104	13.23
	Importance of LTCI	Very unimportant	11	1.40
		Unimportant	19	2.42
		Not so important	138	17.56
		Important	378	48.09
		Very important	240	30.53

*LTCI, long-term care insurance*.

### Insurance awareness, satisfaction with the LTCI policy, and public trust

The participants demonstrated an understanding of available insurance plans. Approximately 70% of the respondents had purchased commercial insurance, 78.5% of the respondents participated in social medical insurance plans, and 45.7% of the respondents were aware that Guangzhou was piloting an LTCI system.

The participants' responses indicated a reasonable level of satisfaction with long-term insurance policies. Forty Four percent of them were satisfied and 20.87% were very satisfied with the way that funds were financed. In addition, participants who were very satisfied (8.65%) and those who were satisfied with reimbursement payment accounted for 65.27% of the sample. Participants who were satisfied (46.18%) and very satisfied (9.16%) with the disability evaluation exceeded 50% of all responses. These figures indicate that the public's satisfaction with the LTCI pilot policy was in a medium to high level.

A total of 62.59% were either willing (49.36%) or very willing (13.23%) to give positive evaluation to the LTCI pilot. Approximately half (48.1%) of the respondents believed that LTCI played an important role and nearly one-third of them (30.5%) believed that it played a very important role, accounting for more than three quarters (78.6%) of the total sample. This finding demonstrated that residents had a high degree of trust in the pilot system of LTCI in Guangzhou.

The results of correlation analysis of insurance awareness, satisfaction, and trust are presented in Table 2. There were significant correlations between any two of the manifest variables.

**Table 2:** Correlation analysis of insurance awareness, satisfaction, and trust.

	**X1**	**X2**	**X3**	**Y1**	**Y2**	**Y3**	**Z1**
X2	74.108^***^						
X3	60.968^***^	33.464^***^					
Y1	10.810^*^	13.542^**^	18.908^**^				
Y2	38.474^***^	21.794^***^	58.648^***^	146.524^***^			
Y3	34.307^***^	12.320^*^	68.809^***^	128.079^***^	359.616^***^		
Z1	57.855^***^	21.356^***^	102.090^***^	155.316^***^	377.887^***^	528.638^***^	
Z2	12.961^*^	26.572^***^	26.342^***^	145.853^***^	109.194^***^	121.437^***^	247.441^***^

*X1, Purchase of private insurance; X2, Participating social insurance; X3, Knowledge of LTCI pilot; Y1, Satisfaction with funding; Y2, Satisfaction with Payment; Y3, Satisfaction with disability evaluation; Z1, Willingness to recommend the LTCI pilot; Z2, Importance of LTCI*.

* *P < 0.05*;

** *P < 0.01*;

*** *P < 0.001*.

### Analysis of ordinal logistic regression results

Table 3 shows the results of the ordinal logistic regression model, including the coefficient estimates, 95% confidence interval, standard errors, and *p*-values. Findings from models 1 and 2 show that residents who had not purchased commercial insurance and did not know that Guangzhou was piloting an LTCI were less likely to give a positive evaluation of the Guangzhou LTCI pilot system. This suggests that they did not believe that the LTCI would be worthwhile. The residents who were less satisfied with the LTCI fund-raising, reimbursement payment, and evaluation processes did not trust the LTCI system.

**Table 3:** Analysis of factors associated with trust in the long-term care insurance system by ordinal logistic regression.

	**Model 1**				**Model 2**			
	**Estimated**	***P*** **-value**	**95% Confidence Interval**		**Estimated**	***P*** **-value**	**95% Confidence Interval**	
	**coefficient**				**coefficient**			
			**Lower bound**	**Upper bound**			**Lower bound**	**Upper bound**
**Insurance awareness**								
Purchase of private insurance (yes = 0)	−0.522	0.004	−0.881	−0.163	−0.323	0.044	−0.638	−0.008
Participating social insurance (yes = 0)	−0.080	0.729	−0.529	0.370	−0.161	0.425	−0.557	0.235
Knowledge of LTCI pilot (yes = 0)	−0.791	0.000	−1.127	−0.455	−0.397	0.007	−0.683	−0.110
**Policy satisfaction**								
Funding = 0	−0.768	0.439	−2.712	1.176	−1.512	0.091	−3.264	0.241
1	−0.554	0.094	−1.203	0.094	−1.389	0.000	−1.986	−0.791
2	−0.965	0.000	−1.437	−0.493	−1.006	0.000	−1.442	−0.570
3	−0.373	0.071	−0.779	0.032	−0.458	0.018	−0.838	−0.077
Payment = 0	−2.961	0.002	−4.862	−1.059	−1.320	0.123	−2.996	0.356
1	−3.124	0.000	−4.080	−2.167	−1.168	0.007	−2.019	−0.316
2	−2.040	0.000	−2.700	−1.379	−1.182	0.000	−1.808	−0.556
3	−0.897	0.003	−1.488	−0.306	−0.590	0.045	−1.168	−0.012
Evaluation = 0	−5.786	0.000	−7.608	−3.964	−2.966	0.000	−4.617	−1.316
1	−2.468	0.000	−3.312	−1.625	−0.708	0.071	−1.477	0.062
2	−1.856	0.000	−2.497	−1.215	−1.000	0.001	−1.608	−0.392
3	−0.681	0.020	−1.255	−0.107	−0.632	0.027	−1.193	−0.071

*Social-economic variables were controlled*.

### Mediating effect of policy satisfaction

The fit indices of the structural equation models are presented in Table 4. The model fitting for the mediating effect of public satisfaction was satisfactory [χ^2^ (16, *N* = 786) = 29.912, *p* = 0.018; TLI = 0.978, CFI = 0.988, RMSEA = 0.033]. The *p*-value of all standardized path coefficients was <0.01, indicating that the relationships among explanatory, response, and the mediating variables were significant.

**Table 4:** Decomposition of mediating effects of policy satisfaction.

**Effect**	**Standardized coefficients**	**Percentage of SC**	**Lower bounds**	**Upper bounds**
Direct	0.201	29.867	0.020	0.358
Indirect	0.472	70.133	0.372	0.737
Total	0.673	100.00	0.573	0.836

*SC, Standardized coefficients. All the coefficients are significant at level 0.01. The response variable is trust*.

The decomposition of direct and indirect effects of each factor in the structural model also proved noteworthy. Table 4 illustrates the direct positive effect awareness had on public trust. Policy satisfaction significantly mediated the indirect effects that awareness had on public trust. For example, both the direct (β = 0.201, *p* < 0.001) and mediated effects (β = 0.472, *p* < 0.001) of awareness on public trust were significant. Among the total effects (β = 0.673, *p* < 0.001), the direct effect accounted for 29.867% of the total effect and the indirect effect accounted for 70.133%. These findings indicate that policy satisfaction partially mediated the relationship between insurance awareness and public trust in the LTCI system.

## Discussion

This study addresses knowledge gap by presenting new empirical evidence on the awareness of and satisfaction with the LTCI policy, and trust in the pilot program in Guangzhou, China. The mediating role of satisfaction in the relationship between the awareness of insurance and trust in the LTCI program was examined. The findings support the proposed hypotheses. This study shows that insurance awareness had a significant direct effect and a significant indirect effect on the participants' trust in the LTCI program, with satisfaction as a mediator. This study's focus on the relationship between insurance awareness and trust in LTCI system provides a novel approach to evaluate the implementation of the LTCI program in China. The results could be used as baseline information for future research on the effectiveness of China's new policies on improving public satisfaction toward public insurance.

The further development of the LTCI in China may bring many potential social and economic benefits. The rising burden of long-term care has been a source of great concern. The LTCI is considered as a national policy that this program can be a viable (49). The LTCI pilot program has made some broad impacts on other programs since its implementation. For example, Shanghai promoted the development of the long-term care industry through the elaboration of action plans to encourage employment in this industry and trained 68,000 frontline workers (50). The city of Nantong was able to reduce medical expenses for families with a disabled person from 162 million yuan ($25.5 million dollars) to 99.7 million yuan ($15.7 million dollars, about 38.5%) since the launch of LTCI pilot (51). Further study of people's attitudes about the implementation of the pilot program are needed because public satisfaction and trust could be important driving forces for the promotion and implementation of the LTCI program in an acceptable and sustainable way (12).

The findings from this study suggest that participants held high expectations for the role that the LTCI program could play. Yet 40% of participants did not provide a positive feedback, which indicates a need to improve public confidence in the system. Trust is a complex concept involving both cognitive and emotional dimensions (34). The cognitive aspect of trust in this case could stem from confidence in the Chinese government's will in implementation of the program in the program's development and promotion (9, 12). Although the Chinese government has been determined to make the LTCI the “sixth insurance” of the social security system, only a small fraction of adult populations have been covered through the pilot program (7). In addition, there are many persistent issues in the supervision and management of the system (9, 13). That is to say, existing problems in the delivery of the long-term care services may affect the public's opinion with regard to the fairness and accessibility of the insurance and hinder people's willingness to evaluate the program in a positive way.

Consistent to the previous studies, the findings from our study show that insurance awareness is correlated with policy satisfaction (21, 33, 52). Participants who exhibited greater levels of understanding of insurance programs expressed a greater satisfaction with the LTCI policy. We also found that satisfaction mediated the effect of health insurance awareness on trust in the LTCI program. This finding adds new knowledge to the literature which study mediates effect of satisfaction between perceived value and trust in public health insurance (21) and between healthcare service quality and trust in care providers (16).

The findings of this study have the potential to inform policy and practice by addressing the ways in which trust in LTCI program could be improved. First, individual' s awareness of insurance should be improved. This study shows that participants' insurance awareness was at a moderate level, primarily reflected in the finding about their limited knowledge toward the LTCI pilot program in Guangzhou. There is a need to improve awareness of the LTCI program *via* a variety of approaches. For example, the government should strengthen the visibility of LTCI through social media, including official website, MicroBlog, WeChat, and community outreach. The LTCI staffs should actively provide explanations for LTCI policy which benefit to the insured to increase their knowledge of LTCI.

Second, there is a need to improve individual's satisfaction with LTCI policy in order to increase the trust toward LTCI system. The survey in this study demonstrates that participants were not highly satisfied with the existing LTCI policy. There are several possible reasons for this. First, the participants did not know much about the LTCI policy in terms of its limited coverage (7, 9). Second, they were not satisfied with the policy terms because of unequal access to LTCI (53). Third, the policy clauses and payment procedures were too complicated and difficult for people to understand. Satisfaction, coupled with trust, could reflect the effectiveness of pilot implementations (44). Therefore, it is important to strengthen policy design of the LTCI program to be more person-centered and optimize the insurance policy by simplifying the reimbursement payment process and evaluation procedures. In addition, measuring residents' perspective of LTCI is essential to know and meet their need for LTCI which would further increase their satisfaction with LTCI.

There are several limitations to this study that needs to be acknowledged. Our sample is limited to one LTCI pilot site. Therefore, the findings from this study could not be generalizable to the general populations in other regions in China. However, we sought to create as much variation in the survey as possible through the recruitment of a large sample of respondents. Further research in other regions and with participants with a variety of demographic profiles would be needed to extend our findings. The second limitation is that the data collection was limited to online survey, due to the social distancing requirement in place due to COVID-19 during the time of the study. Consequently, the study was not able to include people who lacked internet access, smartphones, or computers. The findings of this study should not be considered to be representative. Another limitation was the relatively moderate internal consistency of our self-constructed measuring tool for insurance awareness, satisfaction and trust. We Further research is needed to include instruments with high validity and sensitivity. Finally, due to the cross-sectional survey of the study, we were only able to test the associations across study outcomes of the interest. While our study provides useful new knowledge, future research is needed to conduct relevant longitudinal surveys.

## Conclusion

This study shows that there is a need to improve the residents' satisfaction with and trust toward the pilot of LTCI system in China. Participants' awareness of insurance and satisfaction with LTCI policies significantly affect their trust in the LTCI system. Satisfaction with LTCI policies plays a mediating role in the relationship between insurance awareness and trust. In order to successfully expand LTCI pilot program, there is a need to use multiple approaches to improve insurance awareness and to optimize LTCI policy and promote trust toward LTCI among general population.

## Data availability statement

The raw data supporting the conclusions of this article will be made available by the authors, without unduereservation.

## Author contributions

RP designed the study and secured funding. WZ and RP analyzed data and drafted the manuscript. XD contributes to method and editing. BW contributes to study design and editing. All authors reviewed, provided feedback on, and approved the final manuscript.

## Funding

This work was supported by the National Natural Science Foundation of China (72074055), the Innovation Team Project of Guangdong Provincial Department of Education (2020WCXTD014), and the Scientific Research Project of Guangdong Provincial Department of Education (2018WZDXM004).

## Conflict of interest

The authors declare that the research was conducted in the absence of any commercial or financial relationships that could be construed as a potential conflict of interest.

## Publisher's note

All claims expressed in this article are solely those of the authors and do not necessarily represent those of their affiliated organizations, or those of the publisher, the editors and the reviewers. Any product that may be evaluated in this article, or claim that may be made by its manufacturer, is not guaranteed or endorsed by the publisher.
