# Supplementary material for: Public trust in the long-term care insurance pilot program in China: An analysis of mediating effects
Source: Front Public Health. 2022 Jul 19;10:928745. doi: 10.3389/fpubh.2022.928745 (PMC9343673; doi:10.3389/fpubh.2022.928745)
Supplement: Supplementary file 1 [file Table_1.DOCX]

Appendix

Table S1 Measurements of the latent variables

| Latent variables | Manifest variables | Survey items | Responses |
| --- | --- | --- | --- |
| Insurance awareness | X1 | Have you purchased any commercial pension insurance or commercial health insurance? | No=0, Yes=1 |
|  | X2 | Have you utilized social medical insurance? | No=0, Yes=1 |
|  | X3 | Do you know that there is an LTCI pilot in Guangzhou city? | No=0, Yes=1 |
| Satisfaction with LTCI policies | Y1 | How would you rate your satisfaction with the LTCI funding policy? | Very dissatisfied=0, Dissatisfied=1, Neither satisfied nor dissatisfied=2, Satisfied=3, Very satisfied=4 |
|  | Y2 | How would you rate your satisfaction with LTCI payment policy? | Very dissatisfied=0, Dissatisfied=1, Neither satisfied nor dissatisfied=2, Satisfied=3, Very satisfied=4 |
|  | Y3 | How would you rate your satisfaction with the LTCI disability evaluation processes? | Very dissatisfied=0, Dissatisfied=1, Neither satisfied nor dissatisfied=2, Satisfied=3, Very satisfied=4 |
| Trust in LTCI system | Z1 | How would you rate your willingness to recommend the LTCI pilot program? | Very unwilling=0, Unwilling =1, Not so willing=2, Willing =3, Very willing=4 |
|  | Z2 | How would you rate the degree of importance of the LTCI system? | Very unimportant=0, Unimportant=1, Not so important=2, Important=3, Very important=4 |

Note: X1: Purchase of private insurance; X2: Participating social insurance; X3: Knowledge of LTCI pilot; Y1: Satisfaction with funding; Y2: Satisfaction with payment; Y3: Satisfaction with disability evaluation; Z1: Willingness to recommend the LTCI pilot; Z2: Importance of LTCI.
